# Supplementary material for: Musculoskeletal models of a human and bonobo finger: parameter identification and comparison to in vitro experiments
Source: PeerJ. 2019 Aug 9;7:e7470. doi: 10.7717/peerj.7470 (PMC6690335; doi:10.7717/peerj.7470)
Supplement: Article S1 [file peerj-07-7470-s003.pdf]

Supplemental Article S1: Bonobo dissection and model parameter acquisition

ABSTRACT

This supplemental article describes in more detail how the Bonobo dissection was conducted and how the initial parameters of the bonobo model were acquired.

INTRODUCTION

As mentioned in the main manuscript, initial parameters for the bonobo finger model were needed in analogy to those of the human finger model available from literature (specifically from An et al. (1979)). This comprises the following data:

1. Finger segment lengths for the kinematic description
2. Via point coordinates of each muscle/tendon at each joint
3. Physiological cross sectional areas (PCSA) of each muscle

The respective parameters were obtained from a dissection study performed at the Jan Palfijn Anatomy Lab of the KU Leuven and coordinated by Evie Vereecke. A bonobo specimen (8 years old; female; right arm) was made available by the Antwerp Zoo by Centre for Research and Conservation, Royal Zoological Society Antwerp (KMDA/RZSA) as part of the Bonobo Morphology Initiative 2016.

KINEMATIC DESCRIPTION

Following An et al. (1979), the finger was modelled by three bony segments (distal, middle, and proximal phalanx) interconnected by three joints, namely the distal interphalangeal (DIP) joint, proximal interphalangeal (PIP) joint, and the metacarpophalangeal (MCP) joint. An et al. (1979) defined the kinematics and muscle via points using coordinate systems located proximal and distal to each joint (O_1 to O_6) (Fig. 1). O_2, O_4, and O_6 represent the centres of the DIP, PIP, and MCP joint, respectively, and O_1, O_3, and O_5 are located at the base of each bone. O_0 was added to represent the tip of the finger.

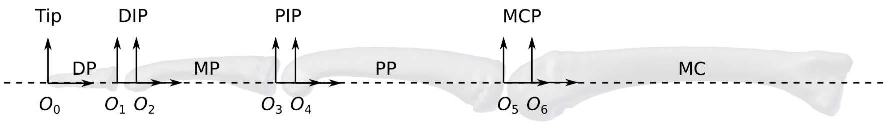

Figure 1. Schematics of the kinematic description following An et al. (1979) defined by the locations of the coordinate systems O_0 to O_6. DIP: distal interphalangeal; PIP: proximal interphalangeal; MCP: metacarpophalangeal; DP: distal phalanx; MP: middle phalanx; PP: proximal phalanx; MC: metacarpal

Coordinate systems O_0 to O_6 were identified using a computed tomography (CT) scan of the bonobo specimen (Discovery CT750, GE Healthcare, USA; voxel size: $0.56 \times 0.56 \times 0.5$ mm^3). The CT image was segmented using the "fill" algorithm of medtool 4.1 (Dr. Pahr Ingenieurs e.U., Pfafstätten, Austria) and smooth triangulated surface meshes were generated using the model maker of 3DSlicer v4.1.0 (Fedorov et al., 2012). Undesired connections between the surfaces of adjacent bones were manually deleted using Blender (v2.64, Blender Foundation, Amsterdam, Netherlands) and the bones were remeshed with the

"remesh modifier" of Blender to ensure all holes of the mesh were closed. The average edge length of the final triangulated meshes of all bones was 0.47 mm.

The joint axes were defined based on the following method (Fig. 2): First, a principal component analysis (PCA) was performed on the vertices of the bone surface mesh to find their longitudinal axes. Second, a plane was defined using the eigenvectors obtained from the PCA which represent the flexion/extension plane or sagittal plane (see Fig. 2). Third, a cylinder geometry was manually fitted to the distal articular curvature of the bone. Fourth, the intersection between the long axis of this cylinder and the flexion/extension plane was computed to find the approximate joint centre of rotation. Steps three and four were repeated five times to investigate their repeatability and led to acceptably low standard deviations below 0.11 mm for each coordinate of the centre of rotation. Finally, the flexion/extension axis was defined by the normal vector of the flexion/extension plane and located at the joint centre of rotation.

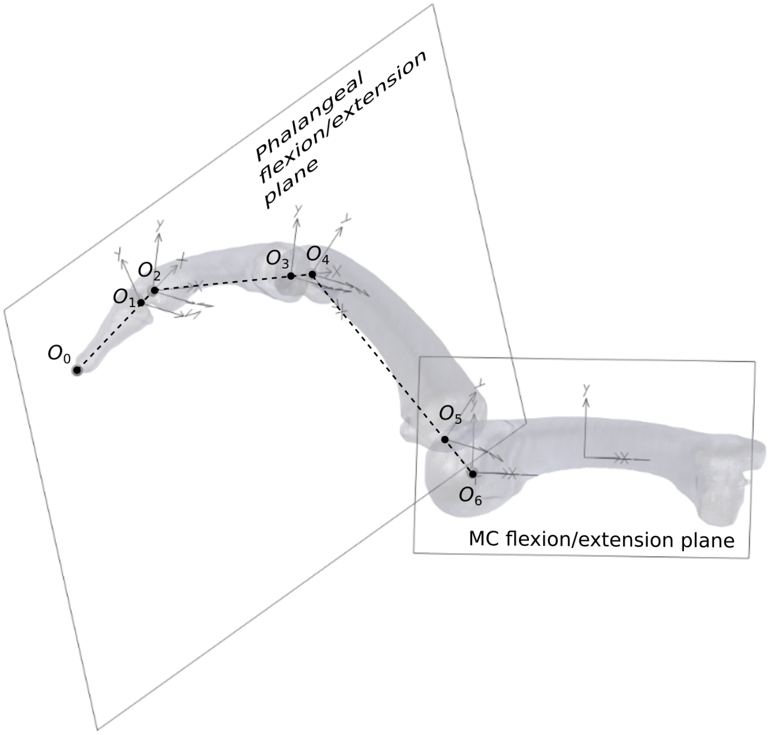

Figure 2. Reconstruction of the kinematic model parameters based on the triangulated surfaces extracted from computed tomography (CT) scans of the bonobo specimen. MC: metacarpal

The above procedure was directly applied to find the flexion/extension axis of the MCP joint using custom Python scripts in Blender. For the interphalangeal joints (PIP, DIP), the normal vectors of the flexion/extension planes of the phalanges were averaged to define a "phalangeal" flexion/extension plane (see Fig. 2). Thus, approximate positions of the joint centres for MCP, PIP, and DIP could be found (i.e. points O_2, O_4, and O_6). The "fingertip" (point O_0 in Fig, 2) was manually selected as the most distal point of the distal phalanx, i.e. the point on the bone surface furthest from O_2.

Points at the base of each each bone (i.e. points O_1, O_3, and O_5) were defined using the following method: First, the line connecting two adjacent joint centres was found (e.g. dashed line between O_0 and O_2 in Fig. 2). Second, the intersection between this line with the respective bone geometry (e.g. distal phalanx bone geometry between points O_0 and O_2) was computed and the most proximal intersection point was selected. By definition, this point also lies on the phalangeal flexion/extension plane. Repeating the above procedure for every bone allowed to find points O_1, O_3, and O_5. All computations were performed using custom Python scripts in Blender.

Finally, two local coordinate systems were defined for each joint (see Fig. 2): The proximal coordinate system is located at the joint centre and the distal coordinate system is located at the base of the bone distal to the joint. The z-axes of those coordinate systems were defined to be perpendicular to the corresponding

flexion/extension plane. The x-axes were defined to be aligned with the connection line between the origin of the coordinate system O_i and point O_{i+1}. The locations of all coordinate systems of the third digit are displayed in Table 1. Values are presented both in absolute numbers and normalized to segment O_2O_3 following An et al. (1979).

Segment	O_0O_1	O_1O_2	O_2O_3	O_3O_4	O_4O_5	O_5O_6
Length (mm)	17.54	3.30	33.91	5.09	49.08	10.00
Normalized Length (-)	0.517	0.097	1.000	0.150	1.447	0.295

Table 1. Segment lengths defining the kinematics of the third digit of the bonobo, both in absolute values and normalized to O_2O_3. See Fig. 1 and 2 for a graphical representation of points O_0 to O_6.

MUSCLE/TENDON VIA POINTS

Following the definition of An et al. (1979), each muscle/tendon path needs to be defined by two via points located proximally and distally with respect to each joint and expressed in proximal and distal coordinate system, respectively. In analogy to the human model, tendons of six muscles of the third digit were included in the bonobo model: flexor digitorum profundus (FDP), flexor digitorum superficialis (FDS), extensor digitorum communis (EDC), lumbrical (LU), radial interosseus (RI), and ulnar interosseus (UI). Additionally, the via points of the extensor mechanism parts, namley radial band, ulnar band, central slip, and terminal slip were required.

The via points were recorded from the bonobo specimen using an electromagnetic six degrees of freedom (DoF) motion tracking system (Patriot, Polhemus, Vermont, USA). First, tendon path points were collected by digitizing points along the tendon at regular intervals (see Fig. 3). In order to obtain tendon path points for all muscles in common coordinate frames, the points were recorded relative to landmarks placed on each bone. These landmarks were defined by four radio-opaque markers (garnet stones attached with bee's wax, see Fig. 3, left) placed on each bone. Thus, the digitized landmarks could be registered to the landmarks identified in the CT scan (Fig. 3, right) and all tendon path points could be transformed into common, bone specific coordinate frames. The landmark registration was performed using the method of Veldpaus et al. (1988) implemented with custom Python scripts.

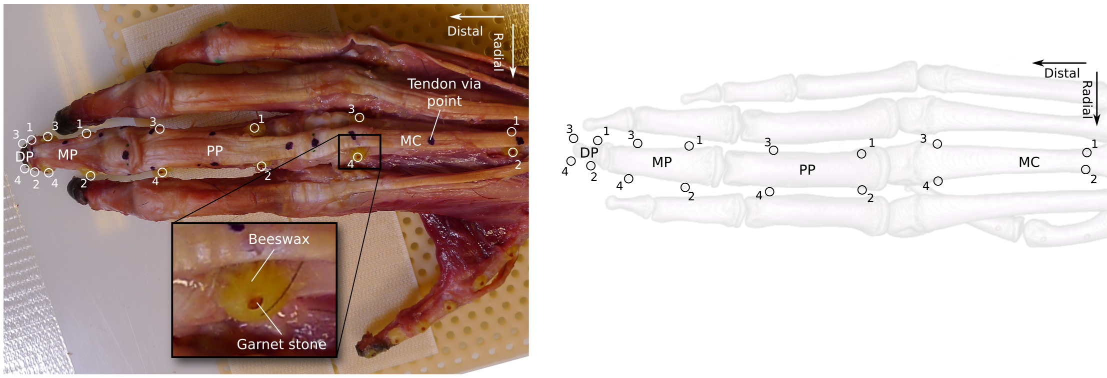

Figure 3. Via point digitization and location of radio-opaque markers during dissection (left) and as identified in the computed tomography scan (right). DP: distal phalanx; MP: middle phalanx; PP: proximal phalanx; MC: metacarpal

Finally, one proximal and one distal point of each tendon relative to each joint which best represented an anatomical constraint (e.g. pulley of a flexor tendon) were chosen and their positions were evaluated in the respective coordinate systems. The resulting via point locations are displayed in Table 2 and Fig. 4.

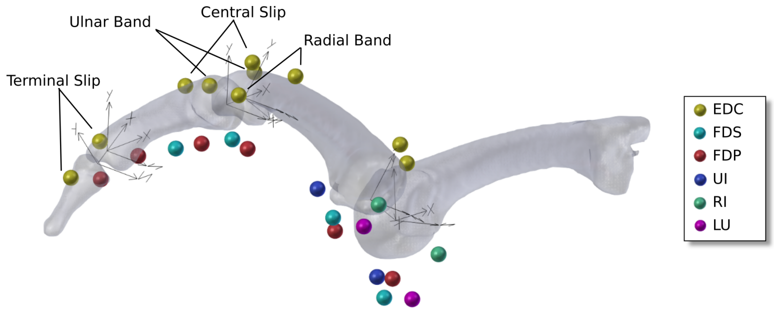

Figure 4. 3D visualization of the tendon via points digitzed during the dissection and registered to the computed tomography (CT) scan. FDS: flexor digitorum superficialis; FDP: flexor digitorum profundus; RI: radial interosseus; UI: ulnar interosseus; LU: lumbrical; EDC: extensor digitorum communis

Joint	Tendon	Distal Point			Proximal Point		
		X	Y	Z	X	Y	Z
DIP	TS	-0.177	0.085	-0.025	-0.024	0.052	-0.035
	FDP	-0.056	-0.057	0.037	0.229	-0.084	0.033
PIP	FDP	-0.304	-0.122	0.091	0.101	-0.244	0.109
	RB	-0.063	0.116	0.139	0.217	0.259	0.082
	UB	-0.099	0.071	-0.223	0.253	0.205	-0.214
	FDS	-0.348	-0.185	-0.057	0.105	-0.224	-0.020
	CS	-0.129	0.142	-0.020	-0.022	0.198	-0.047
MCP	FDP	-0.192	-0.273	0.030	0.051	-0.346	-0.075
	FDS	-0.277	-0.224	0.040	-0.036	-0.437	-0.074
	RI	-0.219	-0.053	0.237	0.165	-0.208	0.164
	LU	-0.160	-0.186	0.170	0.068	-0.455	0.042
	UI	-0.155	-0.079	-0.244	0.086	-0.354	-0.207
	EDC	0.022	0.357	-0.010	0.059	0.318	0.048

Table 2. Proximal and distal tendon via points at each joint, expressed in proximal and distal coordinate systems, respectively. All values were normalized to segment length O_2O_3 as provided in Table 1. FDS: flexor digitorum superficialis; FDP: flexor digitorum profundus; RI: radial interosseus; UI: ulnar interosseus; LU: lumbrical; EDC: extensor digitorum communis; TS: terminal slip; CS: central slip; RB: radial band; UB: ulnar band

MUSCLE PCSA

The physiological cross sectional area (PCSA) of each muscle was computed following the definition of Sacks and Roy (1982) based on the muscle volume V_m, the average muscle fibre length l_m, and the pennation angle ϕ:

$$\mathrm{PCSA} = \frac{V_\mathrm{m}}{l_\mathrm{m}} \cdot \cos\phi \tag{1}$$

V_m, l_m, and ϕ were measured following a previously presented protocol (van Leeuwen et al., 2018). In brief, V_m was assessed by submersion of the muscle belly in a physiological saline solution, l_m was measured using a digital caliper, and ϕ was determined from digital photographs using the software Fiji (Schindelin et al., 2012). l_m and ϕ were measured at three or more sites of the muscle belly and subsequently averaged. If multiple tendons were attached to a muscle belly, the muscle belly volume was divided by the number of tendons attached. Final muscle volumes, averaged fibre lengths, averaged pennation angles, and PCSA values are shown in Table 3.

Muscle	Volume (cm^3)	Fibre length (cm)	ϕ (°)	PCSA (cm^2)
EDC	7.50	6.41	11.00	1.1
FDS	22.50	5.83	23.77	3.5
FDP	28.33	8.97	24.27	2.9
LU*	n/a	n/a	n/a	0.2
RI (EM)**	2.50	2.73	24.70	0.8
RI (PP)**	2.50	1.53	24.57	1.5
UI (EM)**	1.75	1.99	23.57	0.8
UI (PP)**	1.75	1.78	22.20	0.9

Table 3. PCSA data for all muscles of the bonobo specimen. *LU data was not available from the investigated bonobo specimen. Instead, it was averaged from three other specimens previously dissected. **RI and UI consist of two muscle bellies with two tendons, attaching either to the extensor mechanism (EM) or directly to the proximal phalanx (PP). FDS: flexor digitorum superficialis; FDP: flexor digitorum profundus; RI: radial interosseus; UI: ulnar interosseus; LU: lumbrical; EDC: extensor digitorum communis

REFERENCES

An, K. N., Chao, E. Y., Cooney, W. P., and Linscheid, R. L. (1979). Normative model of human hand for biomechanical analysis. *Journal of Biomechanics*, 12(10):775–788.

Fedorov, A., Beichel, R., Kalpathy-Cramer, J., Finet, J., Fillion-Robin, J.-C., Pujol, S., Bauer, C., Jennings, D., Fennessy, F., Sonka, M., Buatti, J., Aylward, S., Miller, J. V., Pieper, S., and Kikinis, R. (2012). 3D Slicer as an image computing platform for the Quantitative Imaging Network. *Magnetic Resonance Imaging*, 30(9):1323–1341.

Sacks, R. D. and Roy, R. R. (1982). Architecture of the hind limb muscles of cats: Functional significance. *Journal of Morphology*, 173(2):185–195.

Schindelin, J., Arganda-Carreras, I., Frise, E., Kaynig, V., Longair, M., Pietzsch, T., Preibisch, S., Rueden, C., Saalfeld, S., Schmid, B., Tinevez, J.-Y. J.-Y., White, D. J., Hartenstein, V., Eliceiri, K., Tomancak, P., Cardona, A., Liceiri, K., Tomancak, P., and A., C. (2012). Fiji: An open source platform for biological image analysis. *Nature Methods*, 9(7):676–682.

van Leeuwen, T., Vanhoof, M. J., Kerkhof, F. D., Stevens, J. M., and Vereecke, E. E. (2018). Insights into the musculature of the bonobo hand. *Journal of Anatomy*, 233(3):328–340.

Veldpaus, F. E., Woltring, H. J., and Dortmans, L. J. (1988). A least-squares algorithm for the equiform transformation from spatial marker co-ordinates. *Journal of Biomechanics*, 21(1):45–54.
