# Supplementary material for: Musculoskeletal models of a human and bonobo finger: parameter identification and comparison to in vitro experiments
Source: PeerJ. 2019 Aug 9;7:e7470. doi: 10.7717/peerj.7470 (PMC6690335; doi:10.7717/peerj.7470)
Supplement: Article S2 [file peerj-07-7470-s004.pdf]

Supplemental Article S2: Musculoskeletal finger model verification with OpenSim

ABSTRACT

This supplemental article presents the verification of the custom Python implementation of the musculoskeletal finger models by comparison to an OpenSim implementation.

INTRODUCTION

In order to ensure that the musculoskeletal finger models as presented in the main manuscript were implemented correctly, their predictions were verified using the frequently used open source musculoskeletal modelling software OpenSim (Delp et al., 2007). In OpenSim, moment arm-based models can be easily implemented and computations of moment arms, muscle forces, and joint loads can be performed. However, tendon bifurcations, such as they occur in the extensor mechanism of the finger, cannot be accounted for. Given the functional significance of the extensor mechanism (Synek and Pahr, 2016), a custom model implementation in Python was preferred over an OpenSim model.

In this supplemental article, a simple finger model including six muscles but no extensor mechanism was implemented in both OpenSim 3.2 and Python (see Fig. 1). All model parameters (bone segment lengths, tendon via points, physiological cross sectional areas) were similar. In the OpenSim model, cylindrical wrapping geometries were added for all tendon segments except the intrinsic muscles (radial and ulnar interosseus, lumbricals) at the metacarpophalangeal (MCP) joint and the radial and ulnar band at the proximal interphalangeal (PIP) joint (see Fig. 1, left). The radii of the wrapping geometries were set to the moment arms of the respective tendon segments in neutral posture. In the Python implementation, wrapping geometries were not directly modelled. Instead, it was assumed that moment arms remain constant if the tendon would naturally wrap around the bone, e.g. an extensor tendon in flexion (see Fig. 1, right). Otherwise, bowstringing conditions were assumed, e.g. a flexor tendon in flexion (also shown in Fig. 1, right). All computations of the Python model followed the descriptions provided in the main manuscript.

MOMENT ARMS

Moment arms of each muscle/tendon at each degree of freedom (DoF) were compared within a predefined range of motion (interphalangeal and MCP joint flexion/extension: -20 to +80 °; MCP joint radial/ulnar deviation: -20 to +20 °). As shown in Fig. 2, the results of the Python implementation were in good agreement with the computations of OpenSim except for a slight mismatch of the extensor digitorum communis (EDC) moment arm for radial/ulnar deviation. The mean absolute error ranged from 0 to 0.03 mm for all but the EDC muscle moment arm in radial/ulnar deviation, which was 0.16 mm.

JOINT TORQUES, MUSCLE FORCES, AND JOINT LOADS

The computation of joint torques from external finger loading, muscle forces, and joint loads were verified with OpenSim using five different test cases (Fig. 3, left column). Test case one was a random posture with loads applied at each finger segment with random magnitude and random orientation. Test cases two to five represented the postures described in the main manuscript, with loads applied at the centre of the distal phalanx and oriented perpendicular to the long bone axis.

The results of the Python model were generally in line with OpenSim, although a slight mismatch of muscle force estimations could be observed (Fig. 3). The error is likely caused by the simplifying assumptions of the wrapping geometries. As expected, this error also propagated to the joint load

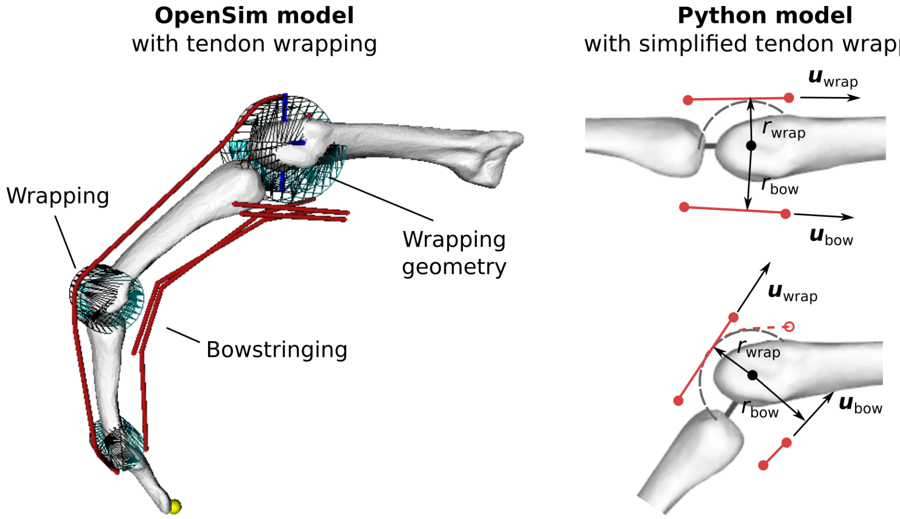

Figure 1. Simplified finger model with wrapping geometries implemented in OpenSim (left) and schematics of simulating tendon wrapping and bowstringing conditions in the Python model (right). r_{bow} and r_{wrap} are the moment arms of two representative tendon segments in bowstringing and wrapping conditions, respectively, and are shown in two different joint postures. $\mathbf{u}_{\mathrm{bow}}$ and $\mathbf{u}_{\mathrm{wrap}}$ are the unit vectors dictating muscle force direction which are necessary for the joint load computation.

predictions, where the error of individual force components ranged from 0 to 0.18 N (0.05 N on average). Relative to the joint load magnitude computed by OpenSim, this means that the error of the force components was within a range of 0 to 2.09 % (0.61 % on average). This relative error was considered acceptable to justify the simplified tendon wrapping assumptions of the Python implementation as a good trade off between accuracy and modelling effort.

REFERENCES

Delp, S. L., Anderson, F. C., Arnold, A. S., Loan, P., Habib, A., John, C. T., Guendelman, E., and Thelen, D. G. (2007). OpenSim: Open-source software to create and analyze dynamic simulations of movement. *IEEE Transactions on Biomedical Engineering*, 54(11):1940–1950.

Synek, A. and Pahr, D. (2016). The effect of the extensor mechanism on maximum isometric fingertip forces: A numerical study on the index finger. *Journal of Biomechanics*, 49(14).

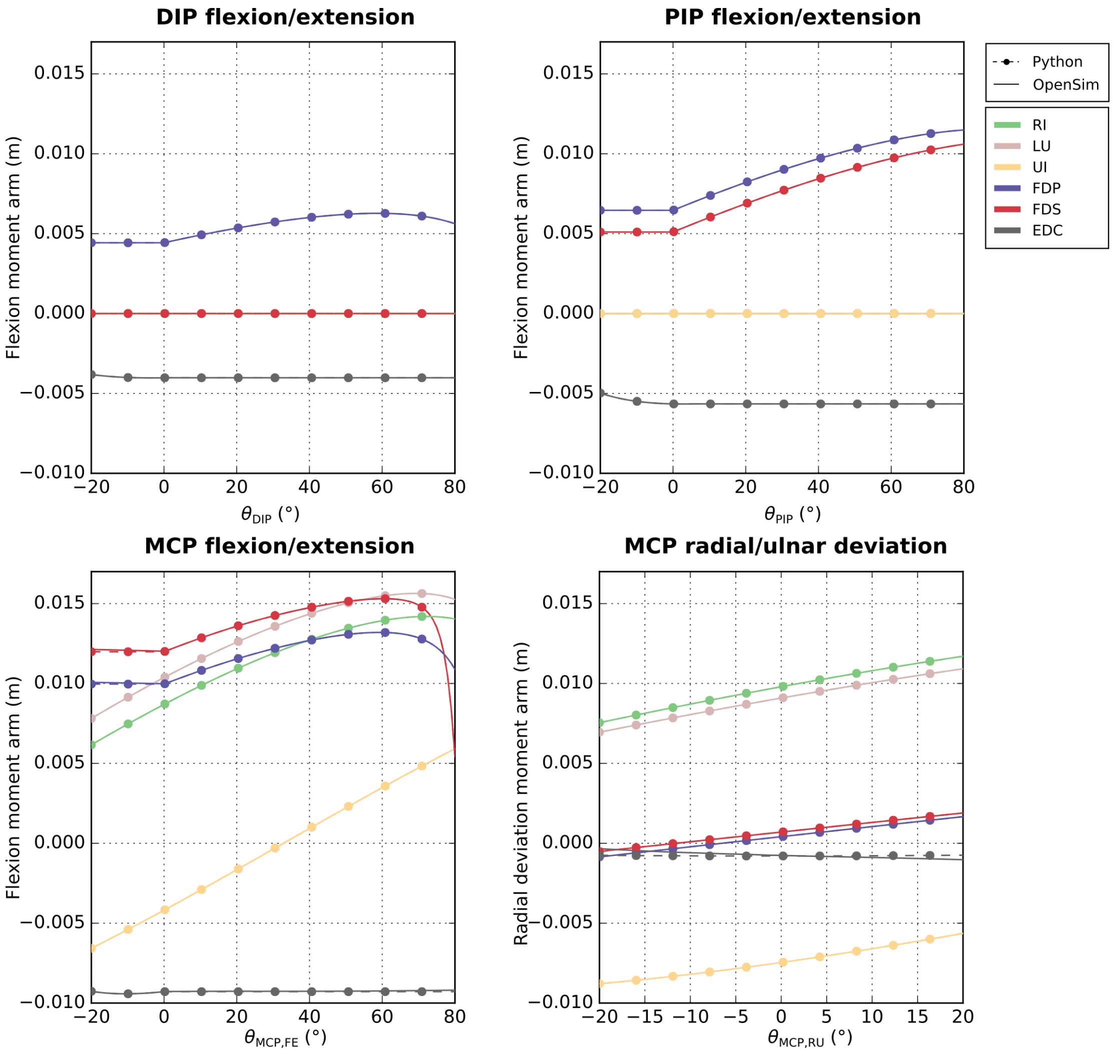

Figure 2. Comparison of moment arms predicted by OpenSim and the custom Python implementation over a large range of motion at all degrees of freedom (θ_i). FDS: flexor digitorum superficialis; FDP: flexor digitorum profundus; RI: radial interosseus; UI: ulnar interosseus; LU: lumbrical; EDC: extensor digitorum communis; DIP: distal interphalangeal; PIP: proximal interphalangeal; MCP: metacarpophalangeal

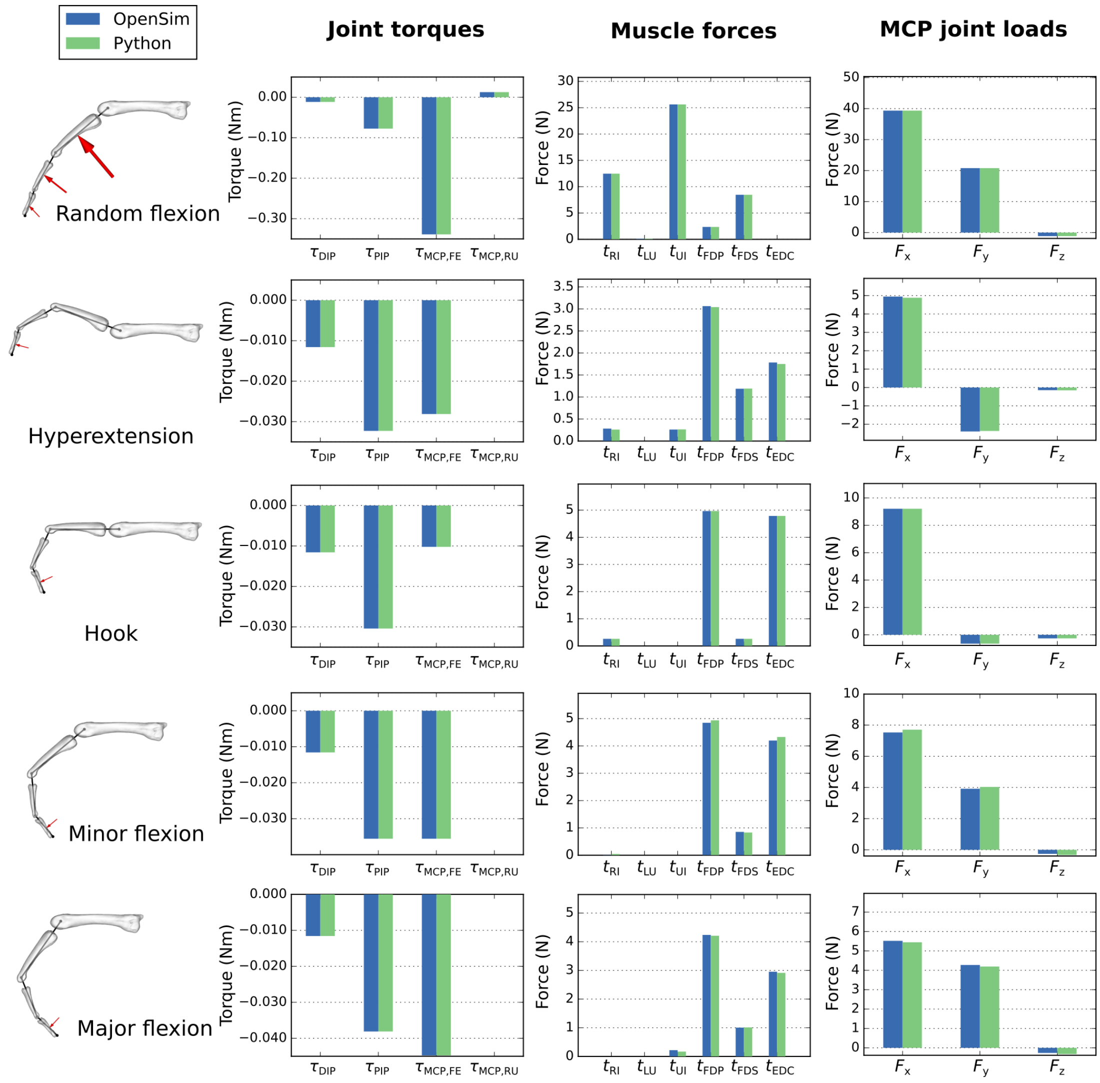

Figure 3. Joint torques resulting from external finger loading at all degrees of freedom (τ_{DIP}, τ_{PIP}, $\tau_{MCP,FE}$, $\tau_{MCP,RU}$), muscle forces (t_{RI}, t_{LU}, t_{UI}, t_{FDP}, t_{FDS}, t_{EDC}), and MCP joint load components (F_x, F_y, F_z) predicted by the OpenSim model and custom Python implementation in five test cases as shown in the leftmost column. FDS: flexor digitorum superficialis; FDP: flexor digitorum profundus; RI: radial interosseus; UI: ulnar interosseus; LU: lumbrical; EDC: extensor digitorum communis; DIP: distal interphalangeal; PIP: proximal interphalangeal; MCP: metacarpophalangeal
