# Supplementary material for: Musculoskeletal models of a human and bonobo finger: parameter identification and comparison to in vitro experiments
Source: PeerJ. 2019 Aug 9;7:e7470. doi: 10.7717/peerj.7470 (PMC6690335; doi:10.7717/peerj.7470)
Supplement: Article S3 — Parameters include bone segment lengths, tendon via point locations, extensor mechanism force transmission fractions, and physiological cross sectional areas of all relevant muscles. [file peerj-07-7470-s005.pdf]

Supplemental Article S3: Final parameters of the musculoskeletal finger models

ABSTRACT

This supplemental article presents the complete set of parameters of both the human and bonobo musculoskeletal finger model after optimization.

SEGMENT LENGTHS

The finger segment lengths describing the kinematics of the finger were directly taken from An et al. (1979) for the human model and from the dissection study (see Supplemental Article S1) for the bonobo model. The segment lengths, normalized to O_2O_3, are shown in Table 1.

For the human, O_2O_3 was set to 23.63 mm as measured and averaged from computed tomography scans of the three cadaveric fingers used in the in vitro experiments. Similarly, O_0O_1 of the human finger was estimated based on these three specimens. For the bonobo finger, O_2O_3 was set to 33.91 mm as measured in the dissection study as described in Supplemental Article S1.

Segment	O_0O_1	O_1O_2	O_2O_3	O_3O_4	O_4O_5	O_5O_6
Human	0.635	0.170	1.000	0.220	1.620	0.370
Bonobo	0.517	0.097	1.000	0.150	1.447	0.295

Table 1. Human and bonobo finger segment lengths, normalized to O_2O_3. The human data was taken from An et al. (1979) except for O_0O_1, which was estimated from computed tomography scans of the three cadaveric specimen used in the in vitro experiments. The bonobo data was directly measured from computed tomography scans as described in Supplemental Article S1.

MUSCLE/TENDON VIA POINTS

Muscle/tendon via points were identified as described in the main manuscript to best match experimentally measured fingertip forces. The final values after optimization for the human and bonobo finger model are presented in Tables 2 and 3, respectively. See An et al. (1979) for the initial values of the human finger model and Supplemental Article S1 for the initial values of the bonobo finger model.

EXTENSOR MECHANISM PARAMETERS

The extensor mechanism parameters were partly fixed and partly identified by the optimization procedure presented in main manuscript. Since radial interosseus (RI) and ulnar interosseus (UI) muscles might insert both in the extensor mechanism and proximal phalanx, the parameters included (1) the fraction of force transmitted to the extensor mechanism e_{EM} (value assumed as fixed based on the PCSA values), and (2) the fraction of force transmitted to the central slip e_{CS} and terminal slip $e_{\mathrm{TS}} = 1 - e_{\mathrm{CS}}$. Lumbrical (LU) and extensor digitorum communis (EDC) muscle parameters were limited to the force transmission fractions e_{CS} and e_{TS} since they insert exclusively into the extensor mechanism. All force transmission fractions after optimization are presented in Table 4.

Joint	Tendon	Distal Point			Proximal Point		
		X	Y	Z	X	Y	Z
DIP	TS	-0.050	0.154	-0.022	0.000	0.158	-0.015
	FDP	0.035	-0.128	0.032	0.293	-0.278	0.033
PIP	FDP	-0.286	-0.287	-0.004	0.416	-0.291	-0.004
	RB	-0.180	0.170	0.227	0.102	0.092	0.242
	UB	-0.180	0.161	-0.247	0.102	0.079	-0.279
	FDS	-0.264	-0.163	0.001	0.332	-0.133	-0.016
	CS	-0.030	0.247	-0.024	0.000	0.231	-0.019
MCP	FDP	-0.232	-0.314	0.023	0.385	-0.518	0.012
	FDS	-0.324	-0.294	0.039	0.487	-0.561	0.019
	RI	-0.312	0.070	0.331	0.269	-0.184	0.471
	LU	-0.370	-0.116	0.328	0.411	-0.598	0.422
	UI	-0.368	0.044	-0.357	0.388	-0.240	-0.358
	EDC	-0.069	0.266	-0.018	0.055	0.409	-0.039

Table 2. Final muscle/tendon via points of the human model, normalized to segment length O_2O_3 and expressed in the proximal and distal coordinate system of each joint (see also An et al. (1979)). FDS: flexor digitorum superficialis; FDP: flexor digitorum profundus; RI: radial interosseus; UI: ulnar interosseus; LU: lumbrical; EDC: extensor digitorum communis; TS: terminal slip; CS: central slip; RB: radial band; UB: ulnar band

Joint	Tendon	Distal Point			Proximal Point		
		X	Y	Z	X	Y	Z
DIP	TS	-0.177	0.084	-0.025	-0.023	0.058	-0.035
	FDP	-0.031	-0.053	0.037	0.228	-0.087	0.033
PIP	FDP	-0.281	-0.085	0.091	0.169	-0.272	0.109
	RB	-0.073	0.105	0.139	0.227	0.254	0.082
	UB	-0.102	0.075	-0.223	0.254	0.208	-0.214
	FDS	-0.344	-0.144	-0.057	0.149	-0.133	-0.020
	CS	-0.129	0.142	-0.020	-0.022	0.202	-0.047
MCP	FDP	-0.304	-0.136	0.030	0.344	-0.428	-0.075
	FDS	-0.362	0.009	0.040	0.306	-0.512	-0.074
	RI	-0.208	0.028	0.237	0.210	-0.019	0.164
	LU	-0.165	-0.148	0.170	0.107	-0.449	0.042
	UI	-0.181	0.178	-0.244	0.378	-0.181	-0.207
	EDC	0.014	0.285	-0.010	0.123	0.283	0.048

Table 3. Final muscle/tendon via points of the bonobo model, normalized to segment length O_2O_3 and expressed in the proximal and distal coordinate system of each joint. FDS: flexor digitorum superficialis; FDP: flexor digitorum profundus; RI: radial interosseus; UI: ulnar interosseus; LU: lumbrical; EDC: extensor digitorum communis; TS: terminal slip; CS: central slip; RB: radial band; UB: ulnar band

Muscle	Human			Bonobo		
	e_{EM}	e_{CS}	e_{TS}	e_{EM}	e_{CS}	e_{TS}
RI	0.500	0.363	0.637	0.353	0.002	0.998
LU	-	0.379	0.621	-	0.456	0.544
UI	1.000	0.381	0.619	0.472	0.350	0.650
EDC	-	0.495	0.505	-	0.255	0.745

Table 4. Force transmission fractions at tendon bifurcations of the extensor mechanism for all intrinsic muscles (RI, UI, LU) and the EDC muscle. RI: radial interosseus; UI: ulnar interosseus; LU: lumbrical; EDC: extensor digitorum communis; TS: terminal slip; CS: central slip

MUSCLE PCSA

Physiological cross sectional areas (PCSA) of the finger muscles were not part of the parameter identification procedure and already presented in the main manuscript. They are reported again in Table 5 to provide a complete parameter description of the finger models in this supplemental article.

**Muscle/tendon**	**Bonobo** PCSA (cm^2)	**Human** PCSA (cm^2)
FDS	3.5	4.2
FDP	2.9	4.1
EDC	1.1	1.7
LU	0.2	0.2
RI (EM)	0.8	1.4
RI (PP)	1.5	1.4
UI (EM)	0.8	2.2
UI (PP)	0.9	0.0

Table 5. PCSAs of the muscles of both the human and bonobo finger. Intrinsics with split tendons inserting into either the extensor mechanism (EM) or the proximal phalanx base (PP) are labelled accordingly. Human finger PCSA values were taken from Chao et al. (1989) and bonobo finger PCSA values were taken from own dissection data (see Supplemental Article S1). EDC: extensor digitorum communis; FDP: flexor digitorum profundus; FDS: flexor digitorum superficialis; RI: radial interosseus; UI: ulnar interosseus; LU: lumbrical; PCSA: physiological cross sectional area

REFERENCES

An, K. N., Chao, E. Y., Cooney, W. P., and Linscheid, R. L. (1979). Normative model of human hand for biomechanical analysis. *Journal of Biomechanics*, 12(10):775–788.

Chao, E., An, K., Cooney, W., and Linscheid, P. (1989). *Biomechanics of the hand: A basic research study*. World Scientific Publishing Company, Singapore.
